# Neurotransmitters: promising immune modulators in the tumor microenvironment

**DOI:** 10.3389/fimmu.2023.1118637

**Published:** 2023-05-05

**Authors:** Luxi Xiao, Xunjun Li, Chuanfa Fang, Jiang Yu, Tao Chen

**Affiliations:** ^1^ Department of General Surgery and Guangdong Provincial Key Laboratory of Precision Medicine for Gastrointestinal Tumor, Nanfang Hospital, The First School of Clinical Medicine, Southern Medical University, Guangzhou, Guangdong, China; ^2^ Department of Gastrointestinal and Hernia Surgery, Ganzhou Hospital-Nanfang Hospital, Southern Medical University, Ganzhou, Jiangxi, China

**Keywords:** neurotransmitter, tumor microenvironment, neuroimmune interaction, cancer immunology, immune modulator

## Abstract

The tumor microenvironment (TME) is modified by its cellular or acellular components throughout the whole period of tumor development. The dynamic modulation can reprogram tumor initiation, growth, invasion, metastasis, and response to therapies. Hence, the focus of cancer research and intervention has gradually shifted to TME components and their interactions. Accumulated evidence indicates neural and immune factors play a distinct role in modulating TME synergistically. Among the complicated interactions, neurotransmitters, the traditional neural regulators, mediate some crucial regulatory functions. Nevertheless, knowledge of the exact mechanisms is still scarce. Meanwhile, therapies targeting the TME remain unsatisfactory. It holds a great prospect to reveal the molecular mechanism by which the interplay between the nervous and immune systems regulate cancer progression for laying a vivid landscape of tumor development and improving clinical treatment.

## 1 Introduction

Cancer, the leading cause of death worldwide, cannot simply be recognized as a single illness but as a manifold group of diseases with diverse causes. As same as blood and lymphatic vessels, nerve fibers transmit signaling molecules and convey nutrients in the tumor microenvironment (TME). Theories of angiogenesis and lymphangiogenesis in tumors thrive over the past decades, but the role of nerves in tumorigenesis is still little known. Similar to the former two, the process tumors stimulate nerve innervation is termed “neoneurogenesis” (1), yet the specific mechanism remains controversial. Some evidence demonstrates that tumor cells can exploit nerve-derived factors to create a favorable microenvironment for tumor survival. Simultaneously, tumors can also stimulate the regeneration of nerve fibers by releasing neurotrophic factors like nerve growth factor (NGF) and axon guidance molecules like netrin-1. Early in 1926, psychosocial factors were demonstrated to be involved in cancer incidence and progression (2). The released neurotransmitters and hormones from neuroendocrine cells transduce the same effects. β-adrenergic agonists or adrenaline showed dose-dependent increases in tumor metastases, while β-adrenergic antagonists and indomethacin synergistically blocked the effects of behavioral stress on lung tumor metastasis. In murine models of cancers, sympathectomy *via* chemical reagents or surgical way and genetic deletion of β2-adrenergic receptors (AR) repressed tumor development in the early stage. Besides, prostate tumor metastases can be abolished by blocking the stromal type 1 muscarinic receptor with medicine or genetic disruption (3), which is the same in a murine model of gastric cancer (4). Sensory neurons can play a role as well. For instance, a model of pancreatic ductal adenocarcinoma has demonstrated that sensory neuron ablation by neonatal injection of capsaicin alleviates tumorigenesis and progression (5).

The immune system is never the minor character in this tug-of-war competition. Stress or depression, the emotional feelings, always do not induce the generation of tumors directly but through psychoneuroimmunology (6). Intricate interplays between neurons and immune cells existed during pancreatitis and modulated inflammation and cancer growth (7). Under chronic stress or depression, a durative-activated hypothalamic-pituitary-adrenal(HPA) axis suppresses the immune response, contributing to tumor development and progression in multiple cancers (6). Specifically, stress and depression were both associated with decreased cytotoxic T cell and natural killer (NK) cell activities and hence influenced immune surveillance of tumors, underlying the increased clinical susceptibility to malignant tumors. In animal models, mental stress, such as swim stress, surgical stress, social confrontation, and hypothermia, led to increased lung metastasis from injected breast cancer cells by suppressing NK cell activity (8–11).

In this review, we focus on discussing the neurotransmitters in the TME and their roles in immune regulation and tumor growth, progression, metastasis, and invasion, as well as their potential opportunities in the clinical treatment of cancer.

## 2 Immunomodulatory neurotransmitters

Immunology has long been studied along with microbiology and pathology. It was generally identified as a self-regulated system by immunologists. Emerging evidence gradually makes it a consensus that the nervous system participates in immune modulation physiologically. As the dominant component, the central nervous system (CNS) regulates immune functions at the whole organism level, moreover, the peripheral nerve endings may also participate in modulating the CNS immune factors or the immune-related neuroendocrine mediators (12). Recently, a noteworthy shift in research focuses happened owing to the discovery that immune cells could produce and release neuroendocrine factors and neuromodulators by themselves (13).

**Figure 1:**
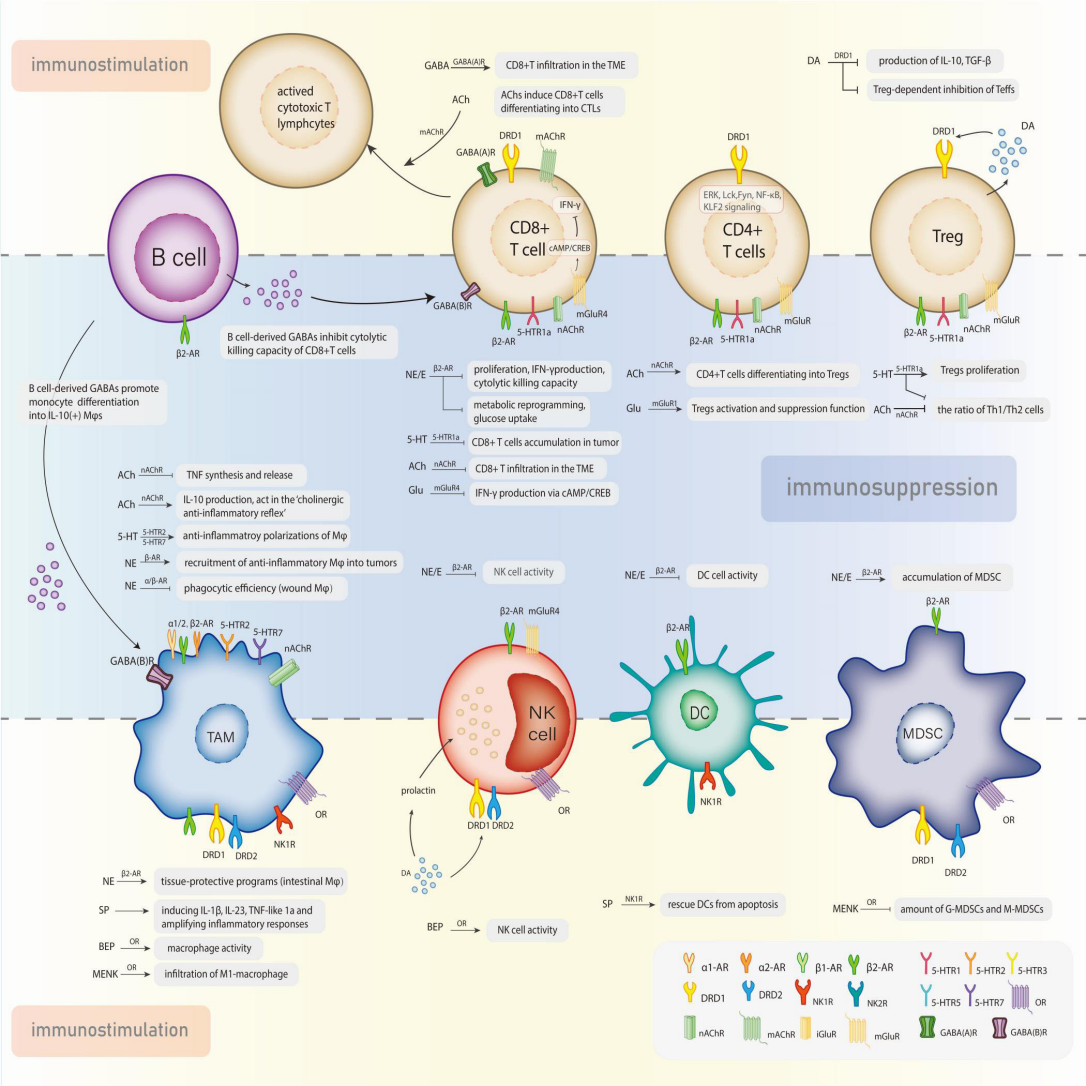
Neurotransmitters exert the dual effects in the modulation of tumor-associated immune cells via specific receptors.

The interactions between the neuroendocrine and immune systems imply a bidirectional circuit where the in-depth mechanism is still obscure. Neurotransmitters, the major modulators in the CNS and perineural system (PNS), have been recognized as potential signaling molecules linking the two major systems for maintaining homeostasis. A series of studies recognized the immunomodulatory function of neurotransmitters that transforms the course of cancer (**Figure 1**). The exact amount of neurotransmitters in total is hard to calculate, but probably over 100, meanwhile their receptors are nearly 1000 (14). Despite the diversity, these molecules can be categorized into two classes: small-molecule neurotransmitters and neuropeptides. Neuropeptides are transmitter molecules composed of 3 to 36 amino acids with neural activity. Amino acids like acetylcholine, glutamate, gamma-aminobutyric acid (GABA), and biogenic amines (including dopamine, norepinephrine, epinephrine, serotonin, and histamine) are much lower in molecular weight and recognized as the classical neurotransmitters. In general, small-molecule transmitters mediate rapid reaction, while neuropeptides are prone to modulating slower responses (15).

### 2.1 Catecholamines

Catecholamines (CAs), the main effectors in the sympathetic nervous system (SNS), are tyrosine-originated biogenic amines and mediate the SNS-induced ‘fight-or-flight’ stress reaction. In response to psychological stress, SNS activation elevates catecholamines levels in circulation *via* the release of epinephrine from the adrenal medulla or norepinephrine spill-over from the neuro-muscular junction of sympathetic nerves (16–18). Generally, an acute SNS activation is beneficial but chronic stress is detrimental as it suppresses the activities of effector immune cells and activates the immunosuppressive cells (19). T cells, as well as macrophages and neutrophils, can synthesize catecholamines themselves and regulate their function in an autocrine/paracrine manner (20). Dopamine (DA), norepinephrine (noradrenaline, NE), and epinephrine (adrenaline, E) are all included.

On the other hand, growing evidence suggests that catecholamines play distinct roles in the regulation of angiogenesis (21–25), which has been clarified as DA inhibits tumor angiogenesis and stimulates tumor immunity while NE and E stimulate angiogenesis and inhibit immune functions in cancer (26).

#### 2.1.1 Norepinephrine and epinephrine

Norepinephrine and epinephrine, known as stress excitatory neurotransmitters, are the main effectors in the sympathetic system. Activated by a stress reaction, they could stimulate muscle contraction, glycogen degradation, airway dilation, and stress-induced tumor progression as well (15).

Norepinephrine and epinephrine perform their functions through α1-,α2- and β-ARs on their target cells respectively. α1 -AR upregulates the intracellular calcium level but α2- AR decreases adenylate cyclase and inhibits intracellular cyclic AMP (cAMP), exerting the opposing functions. There are three subtypes of β-ARs, the G-protein-coupled receptors whose primary function is transmitting information from the extracellular environment to the interior cell and distributing it to the whole body (27). Associated signaling molecules have been summarized as β1- and β2- ARs increase intracellular cAMP by activating adenylate cyclase (27–33).

Both the innate and adaptive immune systems fight against the neoplasms. β-ARs are widely expressed in immune cells, including T lymphocytes, B lymphocytes, NK cells, monocyte/macrophage, and dendritic cells (DCs), of which the activation generally inhibits lymphocyte, NK cell, and DC responses (34, 35). Innate immune cells express the β2-, α1- and α2- ARs, while the β2 subtype is the main receptor on adaptive immune cells, except for Th2 cells (35, 36). Focus has long been on the influence of activated β2-ARs on CD4(+) T cells and B cells. Though CD8(+) T cells express three times the quantity of β2-ARs on CD4(+) T cells, it is still hard to elucidate how the β2-AR-mediated modulation acts in CD8(+) T cells—the backbone of adaptive immunity (37–40). Generally, β-adrenergic signaling significantly suppressed the function of antigen-specific CD8(+)T cells, including their proliferation, interferon-gamma (IFN-γ) production, and cytolytic killing capacity. This T-cell-selective inhibitory effect does not disturb innate lymphocyte responses (41). Moreover, blocked CD8(+) T cell metabolic reprogramming *via* β-adrenergic signaling decreased the glucose uptake of T cells and contributed to stress-induced immunosuppression (42). In addition to suppressing lymphocyte function directly, norepinephrine may downregulate anti-tumor response by favoring the accumulation of immunosuppressive cells, which can be abolished by propranolol in a murine spontaneous model of melanoma (43). As for innate immunity, activated β-AR decreases NK cell activity and permits tumor metastases in an animal model (44, 45). Physiologically, the regulation of NK cell function is closely related to SNS-mediated biological behaviors, such as circadian regulation, exercises, stress, and social engagement (46, 47), and rhythmic NE input to the rat spleen acts as the molecular clock of cellular activity in local NK cells (48). Mobilization and redistribution of NK cells can be motivated by epinephrine in the murine model with regular exercise, which depends on the secretion of IL-6 (49). Endogenous E and prostaglandins orchestrated the inhibition of cytotoxic T-lymphocyte and NK cell responses and promote leukemia progression in rats (50). The affected function of macrophages *via* adrenergic receptors varies under different circumstances. Both physiologic and pharmacologic doses of norepinephrine suppressed wound macrophage phagocytic efficiency through α- and β-AR signaling in a dose-dependent manner (51). With the recruitment of CD11b(+)F4/80(+) macrophages into tumors, the secretion of NE could increase the metastasis of breast cancer cells to distant sites, including the lymph nodes and lungs, without affecting the growth of primary tumors (52). However, intestinal macrophages enhanced tissue-protective programs on luminal bacterial infection *via* activated β2-ARs (53).

#### 2.1.2 Dopamine

Dopamine is an inhibitory stress neurotransmitter in the brain and the precursor for norepinephrine and epinephrine synthesis as well. Though it does not translocate across the blood-brain barrier, dopamine can be detected in the urine, implying its derivation from peripheral tissues. At least three sources of dopamine have been identified: sympathetic neurons, adrenal medulla, and neuroendocrine cells.

Five different seven-transmembrane G-protein-coupled dopamine receptors(DRs) are categorized into two groups: D1 class (D1 and D5) and D2 (D2, D3, and D4) class of receptors on target cells (16, 54). Activated dopamine receptor D1 (DRD1) class increases intracellular cAMP, whereas the dopamine receptor D2 (DRD2) class inhibits intracellular cAMP (54).

Regulation mediated by diverse dopamine receptors is complicated in cancers. In breast and colon cancer preclinical models, dopamine made anti-cancer drugs efficient through an anti-angiogenic effect (55). In gastric cancer, activated DRD2 inhibits insulin-like growth factor (IGF)-I-induced tumor cell proliferation (56). However, the upregulation of DRD1 agitates tumor growth and meanwhile inhibits immunosuppression, but displays an anti-tumor effect in preclinical models (57, 58). DRD1 signaling promoted hepatocellular carcinoma (HCC) cell growth (59). Catecholamines release of CD4(+)CD25(+) regulatory T lymphocytes (Tregs) decreased interleukin-10 (IL-10) and transforming growth factor-β (TGF-β) and inhibited Treg-dependent inhibition of effector-T lymphocytes(Teffs) proliferation, which is selectively reversed by pharmacological blockade of D1-like receptors (60).

SNS has an abundant innervation in the immune system, including most secondary lymphoid organs. Most immune cells or organs express DR, including the thymus and the immune effector cells(e.g., lymphocytes, monocytes, neutrophils, and DCs), suggesting its potential role in modulating the whole immune system (16, 61–65). Both central and peripheral DA have an impact on tumor growth and progression by unbalancing immune homeostasis (66–68). DA can stimulate the peritoneal macrophages, NK cells, and cytotoxic T cells to perform its anti-tumor function (16, 26, 62). Especially, DA has unique and opposite effects on T cell functions, which depends on different DRs level, composition, or dopamine response in various cell types. It was demonstrated that DA activates naïve or resting T cells by D1, D2, D3, and D5 receptors, but inhibits activated T cells by D1, D2, D3, D4, and D5 receptors (69, 70), making their function dynamic. Dopamine itself is a potent activator of resting effector T cells (Teffs) *via* two independent ways: direct Teffs activation and indirect Teffs activation by suppression of Tregs. Dopamine(~10^-8^M) activates resting or naïve Teffs(CD8(+) far outweighs CD4(+)) and affects Th1/Th2/Th7 differentiation *via* ERK, Lck, Fyn, NF-κB and KLF2 signaling cascades (71). However, dopamine in a physiological concentration can significantly inhibit the proliferation and cytotoxicity of CD4(+) and CD8(+) T cells *in vitro*, especially for CD8(+)T cells (72, 73). Except for being an effector, immune cells can be the initiator to secrete DA, such as Tregs (74) aimed at balancing immune homeostasis and influencing the course of disease (75). Activated Tregs produce more dopamine than Teffs in general. In addition, DA can indirectly affect tumor growth by regulating the production and release of prolactin (76–78), which regulates the function of NK cells and lymphokine-activated killer cells (79).

### 2.2 Serotonin/5-Hydroxytryptamine

5-Hydroxytryptamine (5-HT), also named serotonin, is a monoamine neurotransmitter synthesized in the serotonergic neurons within the CNS and the enterochromaffin cells of the intestine (80). More than 90% of the body’s 5-HT is synthesized by the intestine enterochromaffin cells and then stored in platelets. Besides cognitive and behavioral works in the CNS (81), 5-HT also exerts essential roles in peripheral aggregating platelets, provoking immune responses, promoting bone development, regulating insulin secretion, and sustaining systemic energy homeostasis (82, 83). Ovarian cancer progression due to chronic stress was significantly associated with decreased serotonin and inhibited by serotonin/HTR1E signaling (84).

5-HT performs its functions *via* seven different subtypes of receptors (5-HT_1-7_) coupled to multiple signaling pathways. All of the seven belong to the family of G-protein-coupled receptors except for 5-HT_3_—a ligand-gated ion channel. G_i/o_ receptors(5-HT_1_ and 5-HT_5_) coupled to adenylyl cyclase decreased cAMP. G_q/1_ receptors(5-HT_2_) coupled to phospholipase C (PLC) promoted intracellular Ca^2+^ release. G_s_ receptors(5-HT_4_, 5-HT_6_, and 5-HT_7_) coupled to adenylyl cyclase increased cAMP mostly (81, 82).

The multiple effects of 5-HT on depression and the tumor is still far from conclusion. 5-HT itself modulated the macrophage polarization with a sustained anti-inflammatory state predominantly through 5-HT_2B_R and 5-HT_7_R (85). T cell lymphoma invasion and metastasis 2 (TIAM2) promoted colorectal tumorigenesis by maintaining a pro-inflammatory state *via* serotonin-induced immunomodulatory effects (86). 5-HT_1a_R induced an immunosuppressive environment in lung adenocarcinomas patients with depression by activating the p-signal transducer and activator of transcription 3(pSTAT3) and autophagy signaling, as well as upregulating its downstream PD-L1 molecules (87). Specifically, 5-HT_1a_R on T cells is critical for expanding the group of CD4(+)CD25(+)Foxp3(+) Treg cells and reducing the ratio of Th1/Th2 cells, and 5-HT_1a_R on tumor cells is inversely related to cytotoxic lymphocytes activity. Inhibition of platelet-derived peripheral serotonin is associated with decreased pancreatic and colorectal tumor growth in mice, increased CD8(+)T cell influx, and decreased PD-L1 expression in tumors (88).

### 2.3 Acetylcholine

Acetylcholine (Ach), a predominant neurotransmitter of the parasympathetic system, is synthesized and secreted by neurons or nonneuronal cells, such as epithelial cells, mesothelial cells, endothelial cells, immune cells, cancer cells, etc. Apart from the brain, peripheral organs also have an abundant cholinergic innervation, involving a complicated interplay between autonomic nerves and immune cells. Gautron L. et al. found cholinergic fibers in mice gut are close to immune cells, including macrophages, plasma cells, and T cells (89), suggesting a potential role of the cholinergic system in neuroimmune interaction.

Ach receptors can be classified into the nicotinic acetylcholine receptor (nAchR) and the muscarinic receptor (mAchR) (90). Muscarinic receptors provoke immune activities, including lymphocyte mitogenesis, cytotoxic responses, and mast-cell-derived cytokines release. Zimring JC et al. demonstrated M-1 muscarinic receptors improve CD8(+) T cells differentiating into cytolytic T lymphocytes (91). Through nicotinic receptors, acetylcholine inhibited the secretion of tumor necrosis factor (TNF) (92) and stimulated IL-10 production in macrophages in an auto/paracrine manner (93), implying its functional role in immunosuppression. α7nAChR and α4β2nAChR are the evolutionarily oldest nAChRs. α7nAChRs on cytokine-producing macrophages or other immune cells are regarded as the main mediator for the ‘cholinergic anti-inflammatory reflex’’, a prototypical vagus nerve circuit where a memory phenotype T cell population producing acetylcholine was identified (94). Mashimo M et al. identified that α7nAChRs expressed on antigen-presenting cells(APCs) downregulated T cells differentiation by impairing antigen processing, while those expressed on CD4(+) T cells upregulated differentiation into Tregs and Teffs, regulating the intensity of immune responses (95, 96). Activated α7nAChR also mediated PD-L1 expression in normal human bronchial epithelial cells (HBECs) *via* STAT3/NRF2 pathways (97). Another classical nAChR, α4β2nAChR, play opposing roles against α7nAChR in cancer development and progression (98). The two counterparts are in a delicate balance that can be easily broken when the synthesis or release of neurotransmitters or the expression of receptors alters in cancer.

Nonneuronal Ach has been identified as a regulator participating in cell proliferation, differentiation, apoptosis, migration, angiogenesis, and immune response (99–101). Especially, tumor cell-derived Ach can promote tumor progression in an autocrine manner. Wang, ZL et al. found that acetylcholine increased the self-renewal ability of CD133(+) thyroid cancer cells and promoted the expression of PD-L1 *via* the CD133-Akt pathway (102). The pro-tumoral effect of cholinergic signaling was triggered by perineural invasion by sustaining an immunosuppressive environment typical of a reduced CD8(+) T cell infiltration and Th1/Th2 ratio (103). Zhu, P et al. have demonstrated the stimulation of α5nAChR promoted PD-L1 expression and thus induced immune escape *via* the pSTAT3, Jab1 signaling in lung adenocarcinomas (104).

### 2.4 Glutamate

Glutamate, the principal CNS excitatory neurotransmitter, is associated with affective, sensory, motor, and synaptic plasticity, and is also engaged in learning and memory. Abundant glutamate in the TME nourishes cell growth facilitates tumor progression and suppresses anti-tumor immunity. However, some evidence emphasizes that glutamate is also essential for the development and activation of effector T cells to exert anti-tumor function in STK11-/Lkb1-deficient lung cancer (105).

Two classes of glutamate receptors have been identified: the metabotropic receptors(mGluRs) and the ionotropic receptors(iGluRs). According to sequence homology, and pharmacological and intracellular signaling mechanisms, the mGluRs, belonging to the superfamily of GPCRs, are further categorized into three groups. Group I mGluRs(mGluR1 and mGluR5) are coupled to the Gq proteins and their activation stimulates PLC. Whereas, group II(mGluR2 and mGluR3) and III(mGluR4, mGluR6, mGluR7 and mGluR8) are negatively coupled to adenylate cyclase (106). Based on structural similarities, the iGluRs are divided into three subgroups named by the type of synthetic agonist that activates them: N-methyl-D-aspartate(NMDA) receptors, α-amino-3-hydroxy-5-methyl-4-isoxazolepropionate(AMPA) receptors, and 2-carboxy-3-carboxymethyl-4-iso-propenylpyrrolidine(kainate) receptors (107).

Functional iGluRs and mGluRs expressed on normal, tumor, and autoimmune human T cells mediate the activation of many critical cell functions(e.g., adhesion, migration, proliferation), intracellular Ca^2+^ fluxes, and outward K^+^ currents, mainly under a low physiological 10^-8^M to 10^-5^M concentration of glutamate (108). Tumor-derived glutamate leads to peritumoral excitotoxic cell death and thus vacates space for tumor expansion (109–112). Metabotropic glutamate receptor 4(GRM4) plays a novel role in suppressing anti-tumor immunity. Perturbations of GRM4 strengthened the anti-tumor immunity by activating NK, CD4(+) T, and CD8(+) T cells. Specifically, GRM4(-/-) stimulated the IFN-γ production in CD8(+) T cells through cAMP/CREB protein-mediated pathway (113). Various cancers depend on glutamate to an unusual degree for its contribution to metabolic building blocks and the energy supply. Activated mGluR2 and mGluR3 signals promote U87MG human glioma cell growth *in vivo (* 114). Downregulation of glutaminase(GLS)—the critical enzyme converting glutamine into glutamate and regulating glutathione synthesis—diminishes cell-autonomous tumorigenesis in an HCC mouse model (115). GLS1 repression promoted the therapeutic efficacy of anti-PD-L1 therapy with less arginase 1(+) myeloid cells and more CD8(+)/IFNγ(+)/granzyme B(+) T cells, which is also effective in an immune checkpoint blockade(ICB)-resistant mouse model (116). However, GLS2, identified as a p53 target gene, contributes to the p53 tumor suppression *via* its antioxidant and pro-apoptotic function (117).

An antiporter system ${\text{X}}_{c}^{-}$ on the cell surface can import cystine into cells with a 1:1 counter-transport of glutamate, regulating the processes of redox homeostasis and cell growth. Solute Carrier Family 7 Member 11(SLC7A11) or xCT, the light chain subunit of system ${\text{X}}_{c}^{-}$ , serves as the primary transporter (118). Physiologically, imported cystine and intracellular glutamine are converted into cysteine and glutamate respectively, serving as precursors for glutathione(GSH) synthesis, which protects cells from oxidative stress (119). Elevated extracellular glutamate derived from glioblastoma with overexpressed SLC7A11 stimulated the activation and suppressive function of Treg, and the expression of mGlutR1 (120). SLC7A11 repression can be a synergistic anti-tumor mechanism in combination with checkpoint blockade (121). IFN-γ secreted from CD8(+) T cell reduced GSH synthesis in fibroblasts through transcriptional repression of system ${\text{X}}_{c}^{-}$ *via* the JAK/STAT1 pathway, and ultimately abolished the ovarian tumor resistance to platinum-based chemotherapy (122). Similarly, Weimin Wang et al. found that PD-L1 blockade therapy-activated CD8 (+)T cell inhibited SLC7A11 expression, diminished the cystine intake into tumor cells, and hence accelerated tumor cell lipid peroxidation and ferroptosis through IFN-γ (123).

### 2.5 GABA

GABA, a primary inhibitory neurotransmitter in the CNS, is produced from glutamate by the glutamate decarboxylase 1/2 (GAD1/2) enzymes and is catabolized by GABA-transaminase (ABAT). GABA is also widely expressed in the peripheral endocrine organs, including the pituitary, pancreas, gastrointestinal tract, testes, ovaries, placenta, uterus, and adrenal medulla but at a lower level than in the brain (124, 125), and is upregulated in autoimmune diseases and certain solid tumors, such as gastric, pancreatic, and breast cancers (126–129). Three types of GABA receptors include the ionotropic receptors(GABA(A) and GABA(C)) and metabotropic receptors(GABA(B)), inducing different effects on cancer growth (130).

A 2021 publication in Nature has identified B cell-derived GABA promotes monocyte differentiation into IL-10(+) macrophages and limits anti-tumor immunity by inhibiting CD8(+) T cell killer function (131), establishing a suppressive TIME *via* modulating macrophage differentiation. Krummel DAP, et al. demonstrated that benzodiazepines, a drug that can enhance GABA(A)R-mediated anion transport, could depolarize melanoma cells and reduce tumor growth, as well as potentiate radiation and immune checkpoint inhibitor response by provoking direct anti-tumor activity and infiltration of CD8(+) T cell (132). Activated GABA(B) receptor shows contradictory effects on human cancer progression. Baclofen, a GABA(B) receptor agonist, inhibits human HCC growth through the downregulation of intracellular cAMP level and upregulation of p21(WAF1) (133). However, GABA(B) receptor 1 signaling impaired the colorectal tumor cells migration and invasion through blocked EMT and the hippo/YAP1 pathway (134). GABA(B) receptor activated by tumor-derived GABA inhibits GSK-3β activity, enhances β-catenin signaling, and leads to stimulation of tumor cell proliferation and suppression of CD8(+) T cell intratumoral infiltration, suggesting its distinct role of being targeted pharmacologically to reverse immunosuppression beyond its traditional function as a neurotransmitter (135).

### 2.6 Substance P

Substance P(SP), a member of the tachykinin family, is an eleven-amino acid neurotransmitter expressed in CNS or PNS and affects emotional behavior (15). SPs are expressed on the macrophage, neuronal, endothelial, and epithelial cells (136). SP acts on neurokinin-1/2 receptors(NK1/2R), and blocking the neurokinin-1 receptor(NK1R) can inhibit NK1/2R signaling for the treatment of anxiety and depression disorders (137). As the chief receptor for the tachykinin family peptides, NK1R, an inflammation-related G protein-coupled receptor, is widely expressed in the CNS and peripheral tissues. NK1Rs participate in physiological responses such as pain transmission, vasodilation, endocrine and paracrine factors secretion, and cell proliferation (138).

Generally, the stimulatory effects of SPs on immunity consist of accelerating lymphocyte proliferation and the activation of phagocytic cells, bone marrow, and platelets for cytotoxicity (139, 140). DCs, the target of immunotherapy protocols aimed at the stimulation of cellular immune responses, do not always function ex vivo. Signaling *via* NK1R can rescue DCs from apoptosis due to the lack of GM-CSF and IL-4 for ex vivo generation of immune-stimulatory DCs (141). Moreover, the interaction between SP and proinflammatory cytokines modulates the activation of an immune response. NK1R signaling inhibits IL-10 secretion and thus promotes immunostimulatory DCs capable of biasing type 1 immunity (142). To amplify inflammatory responses, SP may function on memory T cells at a local level by inducing the level of IL-1β, IL-23, and TNF-like 1a in monocytes (143).

SP is also a mitogen. Concerning tumor biology, SP stimulates tumor migration in the colon (144) or breast carcinoma cells (145) and induces chemotactic properties in small-cell lung carcinoma cells (146). SP *via* NK1R upregulated toll-like receptor-4 (TLR-4) and contributed to the increase of tumor cell biological activity (147). Anti-SP therapy could strongly suppress cell growth and induce apoptosis in breast, colon, or prostate cancer cell lines and decrease the steady state of Her2 and EGFR (148). NK1R antagonists can also suppress inflammation and metastasis of breast carcinoma cells metastasized into the liver (149). Aprepitant, a kind of NK1R antagonist, prevents macrophages from LPS-induced oxidative stress by reducing the production of ROS and the expression of NOX-4, which may modulate the oxidative state of the TIME (150). Concerning the few available evidence, it is hard to define the exact effects of SP or NKR on anti-tumor immunity now. Clinical administration of NK1R antagonists/agonists still requires abundant examinations.

### 2.7 Opioid peptide

Endorphin, encephalin, and dynorphin, known as endogenous opioids or opioid peptides, are processed from the precursor proopiomelanocortin *via* post-translational cleavage. Leucocyte subsets express proopiomelanocortin (151) and release the products at sites of inflammation, contributing toimmune regulation in pain control (152).

Opioid substances exerted a chief immunosuppressive effect on anti-tumor immunity according to early research (153). However, views differ among the subsequent studies. β-endorphin(BEP), a chemokine for immune cells and small-cell lung carcinoma cells (146), fights against cancers *via* inhibited SNS function and elevates peripheral NK cell and macrophage activities. The effects also involve alterations in the TME, including altered DNA repairs, cell-matrix adhesion, angiogenesis, and epithelial-mesenchymal transition (154). Sarkar, DK et al. transplanted *in-vitro*-generated BEP neurons into the hypothalamic of rats enduring breast carcinogenesis. The BEP neurons-transplanted rats displayed increased immune functions and reduced growth and metastasis of mammary carcinoma, such as activated peripheral NK cells and macrophage, higher anti-inflammatory cytokines, and lower inflammatory cytokines. The opiate antagonist naloxone, beta-receptor agonist metaproterenol, or nicotine acetylcholine receptor antagonist methyllycaconitine can all inactivate NK cells and macrophages, reversing the effects of anti-tumor metastasis (155). Chronic opioid use also alters human CD8(+) T cell subsets balance, including significant decreases in T effector memory RA(+) cells (156).

A clinical investigation on two independent samples involving 1,929 and 1,569 middle-aged women found that the low fasting plasma concentration of encephalin precursor (pro-ENK) is associated with an increased risk of future breast cancer in middle-aged and postmenopausal women (157). According to existing evidence, the function of opioid peptides varies in different cancer, such as methionine enkephalin (MENK) is reported to promote breast carcinoma cells migration (145) but inhibit the cell-cycle process of pancreatic, colon, and head and neck cancer cells (158). Tumor heterogeneity cannot be exclusive of the reason, but no matter the location, the roles of MENK in tumors invariably courted controversy. Multiple pieces of evidence have clarified that MENK exerts anti-tumor effects by enhancing anti-tumor immune response or directly inhibiting tumor cell proliferation (159, 160). In CRC, MENK elevated the M1-type macrophages and T cells infiltration and reduced the groups of myeloid-derived suppressor cells(MDSCs) and M2-type macrophages (159), contributing to a pro-inflammatory state. In a CRC murine model, MENK invigorated immune response by markedly suppressing MDSCs and strengthening T cell activities, thus preventing colon carcinoma progression, which brings light to the development of adjuvant therapy for tumors (160). However, a certain report emphasizes the pro-tumor role of MENK by inhibiting T and B cell proliferation, promoting tumor cell growth, and resulting in the desensitization of lymphocytes *via* opioid receptors (161).

## 3 Clinical opportunities of neurotransmitters in anti-tumor immunity

**Figure 2:**
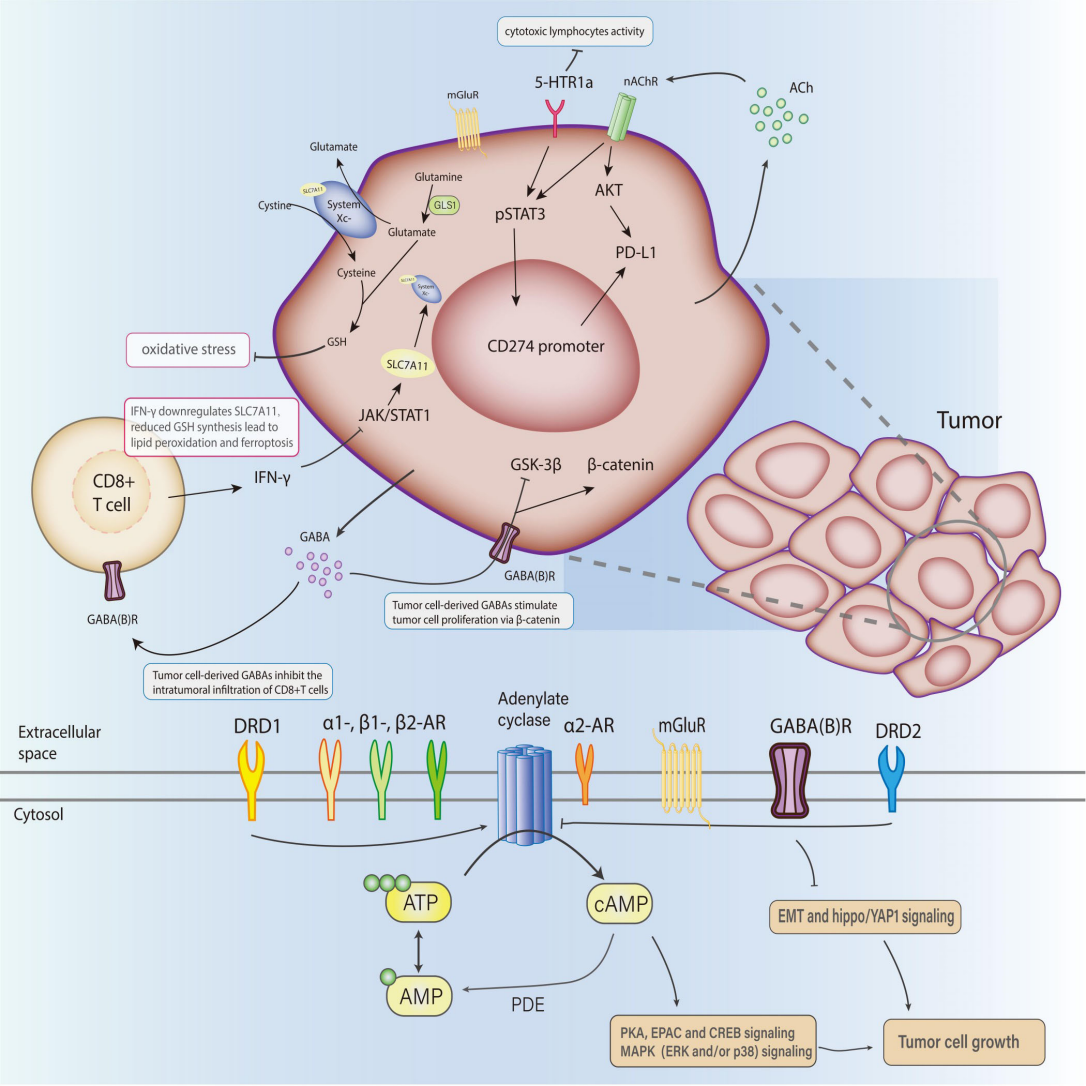
The role of neurotransmitters in the TME and their clinical opportunities.

Immune cells within the TME, named tumor-associated immune cells(TAIs), can defend against proliferation aberrances or conversely induce variations, suggesting their dual role in modulating tumor progression, which generally involves neural stimulation. A highly activated metabolic and energy-consuming state in tumors makes the neuroimmune interaction network more complicated and intensive (**Figure 2**). The administration of β-blockers and antidepressants on cancer patients is initially for other complications besides cancer, such as hypertension, heart disease, stress, or depression. But with the expanded application, these drugs are demonstrated to influence tumor progression or prognosis. Several typical cases are listed below:

### 3.1 β-blockers

β-adrenergic receptors, the chief messengers of sympathetic functions, can activate adenylyl cyclase and accumulate the second messenger cAMP (162) along with accelerated tumor growth (163–165). Overexpressed β-ARs were found in breast and ovarian cancer cells (163, 166), and β2-AR was the dominant subtype on them. According to a large case-control study about prostate cancer patients with simultaneous anti-hypertensive medication, only β-blocker-applied groups have a significant association with reduced cancer risk (167). A cardiovascular patients cohort study showed that the administration of β-blockers resulted in a 49% decrease in cancer risk to never-using relatively (168). Whereas, there is no large population-based case-control study that has confirmed altered risk in invasive breast carcinoma with β-blockers use (169). Activated β2-ARs also enhance the IgE response *via* a PKA-dependent, p38 MAPK-mediated pathway (170). AR regulation is important for cancer vaccine therapy. The role of β2-AR in an effective DC-based cancer vaccination was evaluated in the murine E.G7-ovalbumin(OVA) model and turns out that blocking β2-AR together with the activation of TLR2 at the position of DC inoculation could either promote tolerogenesis or enhance anti-tumor effects (171).

Drug repurposing has been a hot issue in recent years. Concerning the immunomodulatory function mentioned above, β-blockers repurposing may improve the immunotherapies’ efficacy in cancer patients. Several retrospective epidemiological studies have concluded that cancer patients administrated with β-blockers tend to reach better outcomes in prostate, breast, and colorectal cancer (172–175). Similarly, in the murine model, administrating β-blockers can reverse immunosuppression and significantly improve the efficacy of response to checkpoint inhibitor immunotherapy (19). β-blockers can also regulate immune response by modulating the activation of MDSCs and their expression of immunosuppressive molecules(arginase-I and PD-L1). The immunosuppressive effects of MDSCs tend to be alleviated by treating β-blockers or reinforced by β-adrenergic agonists (176). β-blocker for the perioperative treatment of cancer patients abolished the postoperative immune suppression and reduced the risk of tumor metastasis (177–180) by recovering the decreased NK cells cytotoxicity after surgery (181, 182). With this combined method, β-blockers are still warranted because the main factor of surgery-induced recurrence is associated with the postoperative stress response (183).

### 3.2 Antidepressants

Antidepressant drugs are widely used for the clinical treatment of depressive symptoms in cancer patients, modulating tumor growth partly by targeting the immune system (184–186).

Monoamine oxidase A(MAO-A), an enzyme first discovered in the brain, can promote the degradation of monoamine neurotransmitters such as serotonin and dopamine (187). By inhibiting monoamine oxidase to increase available serotonin, MAO inhibitors(MAOIs) enhance anti-tumor T cell activity *via* autocrine serotonin signaling (188) and depolarize alternatively activated immunosuppressive tumor-associated macrophages(TAMs) through the reduction of ROS production (189), suggesting its promising role against tumor-induced immune resistance. With depolarizing TAMs, MAOI treatment could raise the efficacy of other ICB therapies by serving as a TME-engineering therapy. Unfortunately, due to overstimulated serotonin receptors in immunotherapeutic doses, MAOIs may induce aggressive behavioral side effects, which limits their application in anti-tumor therapies (190). Thus, a recent study established a nanoformulation MAOI phenelzine(PLZ) to optimize the administration of MAOIs (191).

Several investigations reveal that SSRIs may inhibit tumor growth through their immune-modulatory actions through the modulation of monoaminergic systems. Fluoxetine, a classic SSRI, significantly inhibits melanoma tumor growth with an increased mitogen-induced T cell proliferation (192) and suppresses the progression of lymphoma *via* restoring NK cell activity and cytotoxic T lymphocyte activity with no noticeable systemic toxicity (193). Fluoxetine also reduced macrophage polarization *in vivo* by reversing tumor-induced oxidative damage and consequent oxidative stress in thymocytes (194, 195). Moreover, fluvoxamine significantly suppressed the migration and proliferation of tumor cells and prompted infiltration of T lymphocytes and M1-type macrophages with reduced PD-L1 molecules in colon cancer murine models (196). Sertraline recovered the T cell stress-induced deficiency by strengthening CD8(+) T cell infiltration, upregulating IFN-γ and Granular enzyme B(GzmB) levels, and reducing PD-1 on CD8(+) T cells, indicating its potential to raise the efficacy of ICB immunotherapy (197).

Tricyclic antidepressant imipramine enhanced autophagy in glioblastoma (GBM) cancer cells and surprisingly reprogrammed immunosuppressive TAMs by suppressing histamine receptor signaling to be immunostimulatory. The combination of imipramine with vascular endothelial growth factor (VEGF) pathway inhibitors orchestrated the infiltration and activation of T cells, supporting anti-PD-L1 therapeutic effects in several GBM mouse models (198).

### 3.3 DRD agonists or antagonists

DA has been demonstrated to play a protective role in cancer patients. According to several epidemiological studies, the compared incidents of cancer between Parkinson’s syndrome(a hypodopaminergic disease) (68) and schizophrenic patients with a probable hyperactive dopaminergic system (199, 200) show the decrease of dopamine are generally followed by higher cancer rates. Contrary to the controversial role of DRD1 in promoting tumor growth but also inhibiting immunosuppression, DRD1 agonists were proven to exert a major anti-tumor effect in several preclinical models (57, 58). Similarly, D1-like receptor agonists can potently inhibit the suppressive function of MDSC, suggesting that dopaminergic signaling tends to modulate tumor growth through strengthening anti-tumor immunity (201). An increased number of breast cancer has been observed in patients treated with DRD2 antagonists (202). However, paliperidone, a DRD2 antagonist, is reported to inhibit GBM growth and decrease the expression of programmed death-ligand 1(PD-L1) in GBM (203), suggesting different roles of DRD2 in different types of cancer.

### 3.4 Cancer immunotherapy and neurotransmitters

Cancer immunotherapy with ICB is based on the inhibition of tumor-mediated immune resistance, instead of directly exerting cytotoxic effects on tumor cells (204). Anti-programmed death-1(PD-1)/programmed death ligand-1(PD-L1) therapy, which circumvents T cell exhaustion due to the immunosuppressive TME by blocking PD-1/PD-L1 checkpoints binding, has been approved by the FDA as a clinical treatment for solid tumors. Considering the major role of T cells in immune defense, the scope of anti-PD-1/PD-L1 therapy is expanding rapidly in clinical practice. However, tumor immune resistance diminishes the efficacy of ICB considerably and becomes an urgent problem to be solved (205).

Neurotransmitters, which prompt immunosuppression, can be potential targets for abolishing immune resistance. For example, cholinergic signaling mainly upregulated the expression of PD-L1 and thus mediated immune escape *in vitro*, inducing an immunosuppressive environment characterized by impaired CD8(+) T cell infiltration and a reduced Th1/Th2 ratio (102, 104). Benzodiazepines, a GABA(A)R activator, potentiated radiation, and ICB response by promoting direct anti-tumor activity and infiltration of CD8(+) T cell (132). Several neural signals show the potential to improve the efficacy of ICB as an adjuvant therapy. In breast cancer, sympathetic denervation surprisingly downregulated the expression of immune checkpoint molecules (PD-1, PD-L1, and FOXP3) (206). In an experimental murine model, the inhibition of β-AR signaling favored an immune-active TME with increased infiltration of CD8(+) T cells, elevated Teffs cell to Tregs cell ratio, and decreased expression of PD-1, which raises the efficacy of anti-PD-1 checkpoint blockade (207). Further research on the involvement of neurotransmitters in TME immunomodulation will be of great interest in improving the efficiency of cancer immunotherapies in the future.

In this review, we discussed the modulatory function of the neurotransmitters in the tumor immune microenvironment (TIME) and their promising application in tumor treatment (**Table 1**). With the further exploration of neuroimmune interactions in the TME, we expect to approach the opportunities for the clinical application of related inhibitors or agonists.

**Table 1:** Immunomodulatory roles of neurotransmitters in the TME.

Neurotransmitter	Receptors	Roles in TME	Supporting details	Clinical opportunities
Catecholamine (CA)	–	DA inhibits tumor angiogenesis and stimulates tumor immunity. NE and E stimulate angiogenesis and inhibit tumor immunity.	An acute SNS activation is beneficial but chronic stress is detrimental generally as it suppresses the activities of effector immune cells and activates the immunosuppressive cells (18). DA inhibits tumor angiogenesis and stimulates tumor immunity while NE and E stimulate angiogenesis and inhibit immune functions in cancer (25).	In breast cancer, sympathetic denervation surprisingly downregulated the expression of immune checkpoint molecules (PD-1, PD-L1, and FOXP3).
norepinephrine (NE), epinephrine (E)	α1-, α2-, β- adrenergic receptors	NE/E triggers the stress-induced tumor progression. NE/E promotes an immunosuppressing environment directly or indirectly. Affected function of macrophages via ARs: opposite evidence.	α1-ARs regulate its function by increasing the intracellular calcium level while α2- AR downregulates adenylate cyclase and thus inhibits cAMP (26). β1-/β2- AR signaling activate adenylate cyclase to increase intracellular cAMP (26–31). Activation of β-ARs usually inhibits lymphocyte responses, NK cell cytotoxicity, and DC functions (33, 34). β-AR stimulation suppresses NK cell activity( related with SNS-mediated biological behaviors) and impairs resistance to tumor metastases in an animal model (45, 46). β-adrenergic signaling significantly suppresses the proliferation, IFN-γ production, and cytolytic killing capacity of antigen-specific CD8(+)T cells and this inhibitory effect is selective to T cells (40), decreasing the glucose uptake of T cells and contributed to stress-induced immunosuppression (41). NE downregulates anti-tumor response by favoring the accumulation of immunosuppressive cells, which can be abolished by propranolol in a murine spontaneous model of melanoma (42). NE in physiologic and pharmacologic doses suppressed wound macrophage phagocytic efficiency through α- and β-AR signaling in a dose-dependent manner (50). With the infiltration of CD11b(+)F4/80(+) macrophages into tumors, NE increased the metastasis of breast cancer cells to distant sites without affecting the growth of primary tumors by indicating M2 macrophage differentiation (51). Intestinal macrophages enhanced tissue-protective programs on luminal bacterial infection via activated β2-ARs (52).	Several retrospective epidemiological studies have concluded that cancer patients taking β-blockers tend to have better outcomes in the prostate, breast, and colorectal cancer (174–177). β-blockers for the perioperative treatment of cancer patients abolished the postoperative immune suppression and reduced the risk of tumor metastasis (179–182) by recovering the decreased NK cells cytotoxicity after surgery (183, 184). inhibition of β-AR signaling in an experimental murine model improved an immunologically active TME with an increased intratumoral frequency of CD8(+) T cells, elevated Teffs cell to Tregs cell ratio, and decreased expression of PD-1, which raises the efficacy of anti-PD-1 checkpoint blockade. β-blockers can regulate immune response by modulating the activation of MDSCs and their expression of immunosuppressive molecules(arginase-I and PD-L1). The immunosuppressive function of MDSCs tends to be mitigated by treating β-blockers or enhanced with β-adrenergic agonists (196).
Dopamine(DA)	D1(D1 and D5) ; D2(D2, D3 and D4)	DAs stimulate anti-tumor immunity. DRD1: agitates tumor growth and inhibits immunosuppression, but ultimately displays the anti-tumor effect DRD2: upregulated in malignant tumors Different DA effects on T cell functions depends on DRs level, composition, or dopamine response in various subtypes. Regulate tumor growth via prolactin release.	DA can stimulate the peritoneal macrophages, NK cells, and cytotoxic T cells to perform its anti-tumor function (15, 25, 61). Immune cells, such as Tregs, can secrete DA to activate immune function (73). DRD1 signaling promoted HCC cell growth (58). Catecholamines release of Tregs led to a reduced production of interleukin-10 (IL-10) and transforming growth factor-β (TGF-β) and suppress its inhibition of Teffs proliferation, which is selectively reversed by blockade of D1-like receptors (59). Inhibition of DRD2 in PDAC cells reduced proliferation and migration, and slowed growth of xenograft tumors in mice (55). DA activates naïve or resting T cells by D1, D2, D3, and D5 receptors, but inhibits activated T cells by D1, D2, D3, D4, and D5 receptors (68, 69), making their function dynamic. Dopamine is a potent activator of resting Teffs by direct Teffs activation or Tregs suppression . Dopamine(~10-8M) activates resting or naïve Teffs and affects Th1/Th2/Th7 differentiation via ERK, Lck, Fyn, NF-κB and KLF2 signaling cascades (79). Dopamine significantly inhibits the proliferation and cytotoxicity of CD4(+) and CD8(+) T cells in vitro under a physiological concentration (71, 72). DA can indirectly affect tumor growth by regulating the production and release of prolactin (84–86), which regulates the function of NK cells and lymphokine-activated killer cells (78).	DRD1 agonists were proven to exert a major anti-tumor effect in several preclinical models (56, 57). D1-like receptor agonists can potently inhibit the suppressive function of MDSC (203). Paliperidone, a DRD2 antagonist, is reported to inhibit GBM growth and decrease the expression of programmed death-ligand 1(PD-L1) in GBM (205).
Serotonin/5-Hydroxytryptamine	5-HT_1_R and 5-HT_5_ R: G_i_/_o_-coupled to adenylyl cyclase and downregulate cAMP. 5-HT_2_R: G_q_/_1_1-coupled to PLC and lead to intracellular Ca^2^+ release. 5-HT_5_R: derives from pseudogene. 5-HT_4_, 5-HT_6_, and 5-HT_7_Rs: G_s_-coupled to adenylyl cyclase and upregulate cAMP mostly.	a potent mitogenic factor for various tumor and non-tumoral cells. 5HT induces the immunosuppressive microenvironment for tumor growth. 5HT mitigates macrophage-induced in vivo immune suppression and T cell apoptosis.	Chronic stress promoted the progression of ovarian cancer cell along with the significantly decreased serotonin, and the effect was inhibited by serotonin/HTR1E signaling (83). TIAM2 provokes a pro-inflammatory immune microenvironment permissive to colorectal tumorigenesis through serotonin-induced immunomodulatory effects (85). 5-HT itself modulated the polarization of macrophages, maintaining an anti-inflammatory state mainly via 5-HTR2B and 5-HTR7 (84). 5-HT1aR induced an immunosuppressive environment in lung adenocarcinomas patients with depression by activating the pSTAT3 and autophagy signaling and upregulating PD-L1 molecules (86). Inhibition of platelet-derived peripheral serotonin is associated with decreased pancreatic and colorectal tumor growth in mice, increased CD8(+)T cell influx, and decreased PD-L1 expression in tumors (87).	Fluoxetine, a classic SSRI, significantly inhibits melanoma tumor growth with an increased mitogen-induced T cell proliferation (194) and suppresses the progression of lymphoma via restoring NK cell activity and cytotoxic T lymphocyte activity with no noticeable systemic toxicity (195). Sertraline recovered the T cell stress-induced deficiency, including strengthening the infiltration of CD8(+) T cells in the TME, upregulating the expression of IFN-γ and GzmB, and reducing the expression of PD-1 on CD8(+) T cells (199). Fluoxetine reduced macrophage polarization in vivo by reversing tumor-induced oxidative damage to macrophages and consequent oxidative stress in thymocytes (196, 197). Fluvoxamine significantly suppressed the migration and proliferation of tumor cells and prompted infiltration of T lymphocytes and M1-type macrophages with reduced expression of PD-L1 in colon cancer murine models (198).
Acetylcholine (Ach)	mAChRs nAChRs (α7nAChR and α4β2nAChR)	Ach upregulates PD-L1 expression and induced immune escape. mAChRs stimulate immune response nAChRs induce immunosuppression mainly	Triggered by perineural invasion, cholinergic signaling favored tumor growth by promoting an immunosuppressive environment characterized by impaired CD8(+) T cell infiltration and a reduced Th1/Th2 ratio (102). Zimring JC et al. demonstrated M-1 muscarinic receptors play a role in the differentiation of CD8(+) T cells into cytolytic T lymphocytes (90). Through nicotinic receptors, acetylcholine inhibited the synthesis and release of TNF (91) and stimulated IL-10 production in macrophages in an auto/paracrine manner (92), implying its functional role in immunosuppression. α7nAChRs on cytokine-producing macrophages and other immune cells have been identified as the main mediator for the 'cholinergic anti-inflammatory reflex’', a prototypical vagus nerve circuit where a memory phenotype T cell population producing acetylcholine was identified (93). Activated α7nAChR mediated PD-L1 expression in normal human bronchial epithelial cells(HBECs) via STAT3/NRF2 pathways (96). α4β2nAChR, play opposing roles against α7nAChR in cancer development and progression (97).	Wang, ZL et al. found that acetylcholine increased the self-renewal ability of CD133(+) thyroid cancer cells and promoted the expression of PD-L1 via the CD133-Akt pathway (101). The stimulation of α5nAChR promoted PD-L1 expression and thus induced immune escape via the pSTAT3, Jab1 signaling in lung adenocarcinomas (103). α7nAChRs expressed on antigen-presenting cells downregulated T cell differentiation by inhibiting antigen processing, while those expressed on CD4(+) T cells upregulated differentiation into Tregs and Teffs, regulating the intensity of immune responses (94, 95).
Glutamate	mGluRs: group I (mGluR1 and mGluR5) coupled to the Gq proteins and their activation stimulates PLC; group II(mGluR2 and mGluR3) groupIII(mGluR4, mGluR6, mGluR7 and mGluR8): negatively coupled to adenylate cyclase iGluRs: NMDARs, AMPARs, kainite receptors	Glutamate facilitates tumor progression, and suppresses anti-tumor immunity. GLS1 suppresses immune therapy and promote tumor; GLS2 contributes to the p53 tumor suppression Glutamate in SLC7A11-high cancer prompts immunosuppression	Tumor-derived glutamate leads to peritumoral excitotoxic cell death and thus vacates space for tumor expansion (108–111). Activated mGluR2 and mGluR3 signals promote U87MG human glioma cell growth in vivo (113). Downregulation of GLS diminishes cell-autonomous tumorigenesis in an HCC mouse model (114). GLS2, identified as a p53 target gene, contributes to the p53 tumor suppression via its antioxidant and pro-apoptotic function (116). Elevated extracellular glutamate derived from glioblastoma with overexpressed SLC7A11 stimulated the activation and suppressive function of Treg, and the expression of mGlutR1 (122).	Perturbations of GRM4 strengthened the anti-tumor immunity by stimulating the IFN-γ production in CD8(+) T cells through cAMP/CREB protein-mediated pathway (112). GLS1 repression enhanced the therapeutic efficacy of anti-PD-L1 therapy, with reduced arginase 1(+) myeloid cells and increased CD8(+)/IFNγ(+)/granzyme B(+) T cells, and delayed tumor growth in an ICB-resistant mouse model (115). SLC7A11 repression can be a synergistic anti-tumor mechanism in combination with checkpoint blockade (123). IFN-γ secreted from CD8(+) T cell reduced GSH synthesis in fibroblasts through transcriptional repression of system Xc- via the JAK/STAT1 pathway, and ultimately abolished the ovarian tumor resistance to platinum-based chemotherapy (124). Weimin Wang et al. found that PD-L1 blockade therapy-activated CD8 (+)T cell downregulated the expression of SLC7A11, impaired the cystine uptake of tumor cells, and hence accelerated tumor cell lipid peroxidation and ferroptosis through IFN-γ (125).
Gamma-aminobutyric acid (GABA)	ionotropic receptors(GABA(A) and GABA(C)): metabotropic receptor(GABA(B)):	GABA(A)Rs: suppress tumor growth and promote anti-tumor immunity GABA(B)Rs( contradictory evidence): impair tumor growth; activate tumor proliferation and promote immunosuppression	Benzodiazepines, a drug that can enhance GABA(A)R-mediated anion transport, could depolarize melanoma cells and reduce tumor growth, as well as potentiate radiation and immune checkpoint inhibitor response by promoting direct anti-tumor activity and infiltration of CD8(+) T cell (134). Baclofen, a GABA(B) receptor agonist, inhibits human HCC growth through the downregulation of intracellular cAMP level and upregulation of p21(WAF1) (135). GABA(B) receptor 1 signaling impaired the migration and invasion of colorectal cancer (CRC) cells by inhibiting EMT and the hippo/YAP1 pathway (136).	B cell-derived GABA promotes monocyte differentiation into IL-10(+) macrophages, an anti-inflammatory subtype, to limit anti-tumor immunity by inhibiting CD8(+) T cell killer function (133), establishing a suppressive TIME via modulating macrophages differentiation. GABA(B) receptor activated by tumor-derived GABA inhibits GSK-3β activity, enhances β-catenin signaling, and leads to stimulation of tumor cell proliferation and suppression of CD8(+) T cell intratumoral infiltration (137).
Substance P (SP)	NK1R, NK2R	SP promotes tumor progression as a mitogen. NK1R signaling activates the immune response by stimulating DCs, T cells, etc. NK1R antagonists inhibit tumor metastases and modulate the oxidative state of TIME.	SP may act locally on memory T cells to amplify inflammatory responses by inducing IL-1β, IL-23, and TNF-like 1a expression from monocytes (145). SP upregulated TLR-4 and contributed to the increase of tumor cell biological activity (149). NK1R signaling inhibits IL-10 secretion and thus promotes immunostimulatory DCs capable of biasing type 1 immunity (144). NK1R antagonists suppress inflammation and metastasis of breast carcinoma cells metastasized into the liver (151). Aprepitant, a kind of NK1R antagonist, prevents macrophages from LPS-induced oxidative stress by reducing the production of ROS and the expression of NOX-4, which may modulate the oxidative state of the TIME (152).	Anti-SP therapy could strongly suppress cell growth and induce apoptosis in breast, colon, or prostate cancer cell lines and decrease the steady state of Her2 and EGFR (150). DCs, the target of immunotherapy protocols aimed at the stimulation of cellular immune responses, do not always function ex vivo. Signaling via NK1R can rescue DCs from apoptosis due to the lack of GM-CSF and IL-4 for ex vivo generation of immune-stimulatory DCs (143).
opioid peptide		BEP inhibits tumor growth involving increased NK cell and macrophage activities. Controversial roles of MENK in cancer and tumor immunity.	β-endorphin(BEP) fights against cancers through the suppression of sympathetic neuronal function, which resulted in increased peripheral NK cell and macrophage activities (156). The BEP neurons-transplanted rats displayed increased immune functions and reduced growth and metastasis of mammary carcinoma, involving increased peripheral NK cell and macrophage activities, increased plasma levels of anti-inflammatory cytokines, and reduced plasma levels of inflammatory cytokines (157). MENK promotes the migration of breast carcinoma cells (147) but inhibits the cell cycle progression of pancreatic, colon, and head and neck cancer cells (160). MENK exerts anti-tumor effects by enhancing anti-tumor immune response or directly inhibiting tumor cell proliferation (161, 162). In CRC, MENK increased the infiltration of M1-type macrophages, CD8(+)T cells, and CD4(+) T cells, and decreased the proportions of G-MDSCs, M-MDSCs, and M2-type macrophages (161, 162). The pro-tumor role of MENK was emphasized by its inhibition of T and B cell proliferation, promotion of tumor cell growth, and the desensitization of lymphocytes via opioid receptors (163).	The opiate antagonist naloxone, the beta-receptor agonist metaproterenol, or the nicotine acetylcholine receptor antagonist methyllycaconitine can all reverse anti-metastatic effects and the stimulation of NK cells and macrophages (157). Chronic opioid use also alters human CD8(+) T cell subsets balance, including significant decreases in T effector memory RA(+) cells (158).

## Author contributions

LX, XL, CF, JY, and TC contributed to the conception and design of the study. LX wrote the first draft of the manuscript. XL, JY, and TC wrote sections of the manuscript. LX, XL, and CF designed the table and figures. All authors contributed to manuscript revision, read, and approved the submitted version.
